# Low-dose IL-2 therapy invigorates CD8^+^ T cells for viral control in systemic lupus erythematosus

**DOI:** 10.1371/journal.ppat.1009858

**Published:** 2021-10-07

**Authors:** Pengcheng Zhou, Jiali Chen, Jing He, Ting Zheng, Joseph Yunis, Victor Makota, Yannick O. Alexandre, Fang Gong, Xia Zhang, Wuxiang Xie, Yuhui Li, Miao Shao, Yanshan Zhu, Jane E. Sinclair, Miao Miao, Yaping Chen, Kirsty R. Short, Scott N. Mueller, Xiaolin Sun, Di Yu, Zhanguo Li

**Affiliations:** 1 Department of Immunology and Infectious Disease, The John Curtin School of Medical Research, The Australian National University, Canberra, ACT, Australia; 2 The University of Queensland Diamantina Institute, Translational Research Institute, Brisbane, Australia; 3 Department of Rheumatology and Immunology, Peking University People’s Hospital, Beijing Key Laboratory for Rheumatism Mechanism and Immune Diagnosis (BZ0135), Beijing, China; 4 Laboratory of Immunology for Environment and Health, Shandong Analysis and Test Center, Qilu University of Technology (Shandong Academy of Sciences), Jinan, China; 5 Department of Microbiology and Immunology, The University of Melbourne, The Peter Doherty Institute for Infection and Immunity, Melbourne, Victoria, Australia; 6 Department of Laboratory Medicine, Affiliated Hospital of Jiangnan University, Wuxi, China; 7 Peking University Clinical Research Institute, Peking University Health Science Center, Beijing, China; 8 School of Chemistry and Molecular Biosciences, The University of Queensland, Brisbane, Australia; 9 Monash Institute of Pharmaceutical Sciences, Monash University, Parkville, VIC, Australia; 10 Peking-Tsinghua Center for Life Sciences, Beijing, China; Johns Hopkins Bloomberg School of Public Health, UNITED STATES

## Abstract

Autoimmune diseases are often treated by glucocorticoids and immunosuppressive drugs that could increase the risk for infection, which in turn deteriorate disease and cause mortality. Low-dose IL-2 (Ld-IL2) therapy emerges as a new treatment for a wide range of autoimmune diseases. To examine its influence on infection, we retrospectively studied 665 patients with systemic lupus erythematosus (SLE) including about one third receiving Ld-IL2 therapy, where Ld-IL2 therapy was found beneficial in reducing the incidence of infections. In line with this clinical observation, IL-2 treatment accelerated viral clearance in mice infected with influenza A virus or lymphocytic choriomeningitis virus (LCMV). Noticeably, despite enhancing anti-viral immunity in LCMV infection, IL-2 treatment exacerbated CD8^+^ T cell-mediated immunopathology. In summary, Ld-IL2 therapy reduced the risk of infections in SLE patients and enhanced the control of viral infection, but caution should be taken to avoid potential CD8^+^ T cell-mediated immunopathology.

## Author summary

Opportunistic infections cause disease exaggeration in patients with autoimmune diseases, representing a leading cause of mortality. Corticosteroids and immunosuppressive therapies often increase the risk of infections. Low-dose IL-2 therapy emerged as a promising new therapy to treat a wide range of inflammatory and autoimmune disorders, but the effect of this therapy to infections has not been systemically evaluated. In this retrospective study, Low-dose IL-2 therapy was found to be associated with the reduced incidence of infection in systemic lupus erythematosus (SLE) patients. In mouse models of influenza A and lymphocytic choriomeningitis virus (LCMV) infection, IL-2 treatment enhanced the effector function of CD8^+^ T cells and accelerated viral clearance but exacerbated CD8^+^ T cell-mediated immunopathology in acute LCMV infection. Our findings show that Low-dose IL-2 therapy might particularly benefit autoimmune disease patients with increased risk of infection due to compromised immunity, such as reduced CD8^+^ T cell function, but caution should be taken to avoid potential CD8^+^ T cell-mediated immunopathology.

## Introduction

One dilemma in clinical practice when treating autoimmune patients is how therapies efficiently control autoimmunity without the pitfall of immunosuppression [1]. In fact, current treatments on autoimmune diseases largely rely on the use of corticosteroids and immunosuppressive medications to effectively control self-reactive immune response by broadly turning down immunity in patients. Numerous studies have found that long-term use of corticosteroids or immunosuppressive drugs can increase the risk of malignancy, opportunistic infections, and osteoporosis in patients, which significantly impact the quality of life and substantially increase the health care cost [2–6]. Therefore, better therapies that can effectively control autoimmunity while keeping the immune-competent and protecting from infection and malignancy are urgently needed [1].

Infection and autoimmunity can reinforce each other in autoimmune diseases. Firstly, infections can trigger the onset of autoimmunity and cause subsequent flares [7]. During infections, tipping the balance away from immune tolerance and homeostasis initiates immune activation that recognizes not only foreign but also self-antigens. The latter might catalyze an immune cascade eventually leading to self-destruction of the host tissues and organs. The priming of cross-reactive T cell clones, which is known as “molecular mimicry”, and bystander activation of non-pathogen specific T cells are considered to promote infection-induced autoimmunity [8,9]. On the other hand, immunosuppressive treatments in autoimmune patients can impair protective immunity which profoundly increases the risk of opportunistic infections in these patients [10,11]. Glucocorticoid is the most widely used immunosuppressive drug, received by more than 1% of the UK and US population for autoimmune and inflammatory diseases and organ transplantation [12]. It significantly increases the susceptibility of patients to invasive fungal, lower respiratory tract infection and local candidiasis [12,13]. Using rheumatoid arthritis as an example, clinical studies showed that glucocorticoid is a strong risk factor for increased infection [14,15], with those receiving over 10 mg of glucocorticoid per day increasing their risk of hospitalization from 6.78% to 13.25% [16]. Infection counts for around 30% to 50% of morbidity and mortality of systemic lupus erythematosus (SLE) patients [17–19] with the report showing that infection is the second leading cause of death for SLE patients, estimated as 18% [20]. More recently, infection was considered as the leading cause of death for SLE patients in multiple cohorts. Immunosuppressive treatments such as cyclophosphamide and glucocorticoids, not only increase the risk of opportunistic infections but also result in severe organ damages [19,21–25]. Other biologics such as anti-CD20 Rituximab for SLE or TNF-α inhibitors for rheumatoid arthritis were reported to associate with increased risks of infection in patients [26–29]. Therefore, it is necessary to evaluate the risk of infection for new immunomodulatory therapies for autoimmune diseases.

Low-dose interleukin-2 (Ld-IL2) therapy emerges as a promising new therapy to treat a wide range of inflammatory, autoimmune and alloimmune disorders such as SLE, hepatitis C-induced vasculitis, graft-versus-host disease (GVHD) [1,30]. The broad application of Ld-IL2 therapy is considered to be underpinned by the multiple mechanisms of its action, not only boosting the function of regulatory T (T_REG_) cells [31] to strengthen immune tolerance and homeostasis but also suppressing effector IL-17-producing helper T (T_H_17) [32] and follicular helper T (T_FH_) cells [33–35] to ameliorate inflammation and autoimmunity in lupus patients [30,36]. In another phase 1 and 2a clinical trial, lupus patients who received Ld-IL2 therapy also showed improved disease activity evaluated by the Safety of Estrogens in Lupus National Assessment-Systemic Lupus Erythematosus Disease Activity Index (SELENA-SLEDAI) and increased frequency of CD25^+^Foxp3^+^ T_REG_ cells [37]. Similarly, most clinical trials of Ld-IL2 therapy in other autoimmune diseases measured CD4^+^ T cells subsets, particularly T_REG_ cells as the primary endpoints, including GVHD [38,39], Vasculitis [40], type 1 diabetes [41,42] and Sjögren’s syndrome [43].

In addition to IL-2’s selective regulation of different CD4+ T cell subsets [44], it has been well-characterized that the paracrine and autocrine production of IL-2 is critical in supporting the activation and proliferation of CD8^+^ T cells and promote their memory formation. Considering a central role of CD8^+^ T cells in eliminating infected cells and controlling intracellular infections [45], Ld-IL2 therapy might not cause the immunosuppressive effects of glucocorticoids or other immunosuppressive drugs observed in autoimmune patients upon treatment, suggesting a potentially ideal scenario whereby Ld-IL2 therapy reinstates immune tolerance and homeostasis without inducing significant immunosuppression. This notion is supported by the observation of a previous clinical trial showing that Ld-IL2 ameliorated hepatitis C virus-induced vasculitis without perturbing virus control [40]. More recently, in a randomized, double-blind, placebo-controlled trial of Ld-IL2 therapy in SLE, no serious infection was observed in the IL-2 group, in contrast to two cases in the placebo group, again supporting this notion [46,47].

Therefore, we were inspired to formally evaluate the relationship between Ld-IL2 therapy and infection risk in autoimmune diseases by conducting a retrospective cohort study by comparing the incidence in infection between a large cohort of SLE patients with standard treatment plus Ld-IL2 therapy and a control cohort with standard treatment only. To verify the observation in the human study, we further investigated the immunoregulatory function of Ld-IL2 therapy using multiple mouse models of viral infection to determine its benefits and potential adverse effects.

## Results

### Characterization of SLE patients

**Fig 1:**
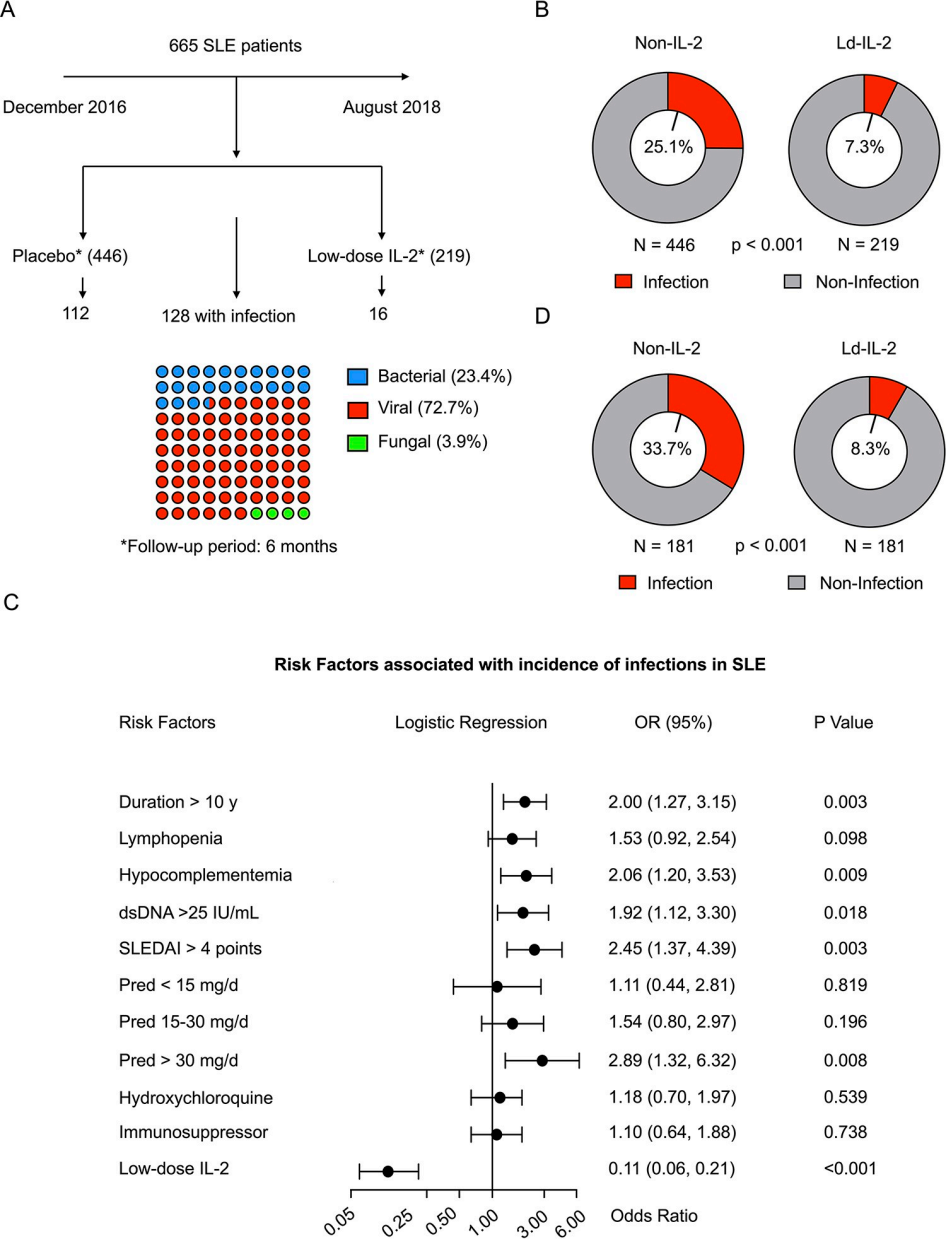
Ld-IL2 therapy is a protective factor associated with infection in SLE patients. This study includes 665 Systemic lupus erythematosus patients that were admitted in Peking University People’s Hospital between 2016 to 2018 (A-D). Among these patients, 219 were treated with Ld-IL2 and immunosuppressive therapy while 446 were treated only with immunosuppressive therapy (steroids or immunosuppressive agents). The follow-up periods were 6 months. (A) Schematic for the clinical study with 128 infections caused by bacteria, virus and fungi recorded. (B) Incidence rates of infection were calculated between patients with or without Ld-IL2 therapy (*n* = 665). (C) Multivariate analysis was conducted to evaluate the risk factors associated with infection in SLE patients, presented as logistic regression vs Odds ratio. (D) Propensity score matching method was applied to generate 181 matched pairs of patients with or without Ld-IL2 therapy, followed by comparison of the incident rates of infection in these two matched cohorts (*n* = 181, each). Fisher’s exact test or Mann-Whitney U-tests were performed to compare the differences. *p <0.05, **p <0.01.

To evaluate the incidence of infection in SLE patients treated with Ld-IL2 therapy, we retrospectively assessed 665 SLE patients admitted to Peking University Peoples Hospital (Beijing, China) from December 2016 to August 2018, including 446 patients treated with standard of care (corticosteroids and conventional immunosuppressive agents) as non-IL-2 group and 219 patients treated standard of care plus 3 cycles of Ld-IL2 as IL-2 group (**Fig 1A**). The characteristics of these patients are shown in **Table 1**. Patients in these two groups were age and sex matched. The baseline median disease activity index of SLEDAI-2K (Systemic Lupus Erythematosus Disease Activity Index 2000) of IL-2 group was higher than that of non-IL-2 group (5 v.s. 2 points, P-value < 0.001), in line with a higher frequency of active lupus nephritis (LN) in IL-2 group (33.3%, 73/219) than non-IL-2 group (12.3%, 55/446) (P-value < 0.001) (**Table 1**). All possible patients in our hospital received Ld-IL2 therapy from December 2016 to February 2018 and were recruited in this retrospective study. This patient cohort had poor response to standard therapy or had exacerbated conditions. In addition, more patients in IL-2 group had a history of corticosteroid treatment (90.4%, 198/219 v.s. 84.3%, 376/446, P-value = 0.031) with significantly higher doses (**Table 1**). More severe disease and elevated treatment of corticosteroids in IL-2 group than non-IL-2 group suggested a higher risk of infection in the former.

**Table 1:** Baseline clinical characteristics of patients with SLE.

Variables	Ld-IL2 (n = 219)	Non-IL-2 (n = 446)	*P* value
Gender, Female^a^	189 (86.3)	385 (86.3)	-
Age, year^a^	34 (27.47)	34 (26,48)	-
Duration, year	7 (3,12)	6 (3,11)	0.315
Comorbidities			
Nephritis	73 (33.3)	55 (12.3)	<0.001***
Diabetes mellitus	11 (5.0)	17 (3.8)	0.465
Chronic pulmonary disease	6 (2.7)	12 (2.7)	0.971
Treatments			
Prednisone	198 (90.4)	376 (84.3)	0.031*
Baseline prednisone, mg	20 (10,45)	7.5 (5,12.5)	<0.001***
< 15 mg/d	81 (37.0)	297 (66.6)	<0.001***
15–30 mg/d	47 (21.5)	32 (7.2)	<0.001***
> 30 mg/d	70 (32.0)	47 (10.5)	<0.001***
Hydroxychloroquine	160 (73.1)	328 (73.5)	0.895
Cyclophosphamide	22 (10.0)	36 (8.1)	0.397
Mycophenolate mofetil	90 (41.1)	179 (40.1)	0.812
Cyclosporine	28 (12.8)	61 (13.7)	0.751
Tacrolimus	12 (5.5)	20 (4.5)	0.573
Azathioprine	20 (9.1)	30 (6.7)	0.269
SLEDAI-2k	5 (2, 9)	2 (0, 5)	<0.001***
≤ 4 points	102 (46.6)	329 (73.8)	<0.001***
5–9 points	63 (28.8)	86 (19.3)	0.006**
10–14 points	52 (23.7)	30 (6.7)	<0.001***
≥ 15 points	2 (0.9)	2 (0.4)	0.602
Infection	16 (7.3)	112 (25.1)	<0.001***

Data expressed as median (IQR) and n (%) and compared by Fisher’s exact test or Mann-Whitney *U*-tests.

***P<0.001

**P<0.01

*P<0.05.

^a^ Cohorts were matched for age and sex.

Ld-IL2, low-dose interleukin 2; SLEDAI-2k, Systemic lupus erythematosus disease activity index-2000.

### Ld-IL2 therapy reduces incidence of infection in SLE patients

Among these 665 patients, 124 (18.6%) patients reported 128 infection episodes. The rate of infection in the IL-2 group (7.3%, 16/219) was more than 3-fold lower than that of the non-IL-2 group (25.1%, 112/446) (*P*-value < 0.001, **Fig 1B**). In total, 30 cases of bacterial (23.4%), 93 cases of viral (72.7%) and 5 cases of fungal (3.9%) infections were recorded (**Fig 1A** and **Table 2**). Compared to the non-IL-2 group, patients receiving Ld-IL2 treatment showed reduced bacterial infection (1.4% 3/219 v.s. 6.1%, 27/446, P-value = 0.006), virus (5.9%, 13/219 v.s. 17.9%, 80/446, P-value < 0.001) but no difference was observed to fungal infections which was rare in our cohort, respectively (**Table 2**). IL-2 group had lower incidence of infection than non-IL-2 group in upper respiratory tract infection (4.6%,10/219 v.s. 13.9%, 62/446, *P*-value < 0.001) and to herpes zoster infection (0%, 0/219 v.s. 2.9%, 13/446, *P*-value = 0.007) (**Table 2**).

**Table 2:** Infectious agents identified in patients with SLE.

Type	Organism	Ld-IL-2 (n = 219)	Non-IL-2 (n = 446)	*P* value
Bacterial	Gram (+)	1 (0.5)	9 (2.0)	0.178
	Gram (-)	1 (0.5)	11 (2.5)	0.116
	Mycoplasma	0 (0.0)	1 (0.2)	1.000
	Unknown type	1 (0.5)	6 (1.3)	0.435
	Total	3 (1.4)	27 (6.1)	0.006**
Viral	Herpes zoster	0 (0.0)	13 (2.9)	0.007**
	Cytomegalovirus	3 (1.4)	1 (0.2)	0.107
	Influenza	0 (0.0)	4 (0.9)	0.308
	Viruses related to URTI	10 (4.6)	62 (13.9)	<0.001***
	Total	13 (5.9)	80 (17.9)	<0.001***
Fungal	PCP	0 (0.0)	1 (0.2)	1.000
	Candida	0 (0.0)	3 (0.7)	0.555
	Aspergillus	0 (0.0)	1 (0.2)	1.000
	Total	0 (0.0)	5 (1.1)	0.177
Total	—	16 (7.3)	112 (25.1)	<0.001***

Data expressed as n (%) and compared by Fisher’s exact test.

***P<0.001

**P<0.01.

IL-2, interleukin 2. URTI, Upper respiratory tract infection; PCP, Peumocystis Carinii Pneumonia.

### Ld-IL2 therapy is a protective factor of infection in SLE

To identify risk factors of infection in patients with SLE, a stepwise logistic regression was performed (**Table 3 and Fig 1C**). Specifically, we separated all 665 patients into patients with infection (infected group) or without (Non infected group) and compared their baseline characteristics (**Table 3**). In the univariate analysis, compared to the patients in the non-infected group (n = 537), patients with infection (n = 128) had longer disease duration (> 10 years), higher incidence rate of lymphopenia and hypocomplementemia. Moreover, increased proportion of SLE patients showed positive anti-dsDNA antibody and higher active disease (SLEDAI-2k > 4 points) than those patients without infection. Intriguingly, lower percentage of SLE patients with infection received Ld-IL2 therapy compared to those without infection (16, 12.5%, v.s. 203, 37.8%, P-value < 0.001) (**Table 3**). In the multivariate analysis, infection associated factors included clinical features of long disease history (disease duration > 10 years, OR = 2.00, 95% CI 1.27 to 3.15, P-value = 0.003) and high disease activity (SLEDAI > 4 points) (OR = 2.45, 95% CI 1.37 to 4.39, P-value = 0.003), hypocomplementemia (OR = 2.06, 95% CI 1.20 to 3.53, P-value = 0.009), anti-dsDNA antibody (OR = 1.92, 95% CI 1.12 to 3.30, P-value = 0.018), and corticosteroid therapy (dose > 30 mg/d, OR = 2.89, 95% CI 1.32 to 6.32, P-value = 0.008) (**Fig 1C**). In contrast, Ld-IL2 therapy was identified as a factor negatively associated with infection incidence (OR = 0.11, 95% CI 0.06 to 0.21, P-value < 0.001) (**Fig 1C**).

**Table 3:** Risk factors of infection in SLE patients with univariate analysis.

Variables	Category	Infected (n = 128)	Non infected (n = 537)	*P* value
Age	—	34 (28.5, 46)	35 (28, 50)	0.614
Sex	Male	18 (14.1)	73 (13.6)	0.167
	Female	110 (85.9)	464 (86.4)	
Disease duration	≤ 10 years	70 (54.7)	359 (66.9)	0.01*
	> 10 years	58 (45.3)	178 (33.2)	
Lymphopenia	Yes	95 (74.2)	294 (54.7)	<0.001***
Hypocomplementemia	Yes	92 (71.9)	245 (45.6)	<0.001***
Anti-dsDNA antibody	≤ 25 IU/mL	42 (32.8)	322 (60.0)	<0.001***
	> 25 IU/mL	86 (67.2)	215 (40.0)	<0.001***
Nephritis	—	25 (19.5)	103 (19.2)	0.928
Chronic kidney disease	—	54 (42.2)	259 (48.2)	0.218
Diabetes mellitus	—	3 (2.3)	25 (4.7)	0.242
Chronic pulmonary disease	—	4 (3.1)	14 (2.6)	0.746
Prednisone	Yes	117 (91.4)	457 (85.1)	0.062
Prednisone dose	< 15 mg/d	71 (55.5)	295 (54.9)	0.913
	15–30 mg/d	26 (20.3)	65 (12.1)	0.015*
	> 30 mg/d	20 (15.6)	97 (18.1)	0.515
Hydroxychloroquine	—	98 (76.7)	390 (72.6)	0.365
Immunosuppressive	—	97 (75.8)	394 (73.4)	0.577
Cyclophosphamide	—	13 (10.2)	45 (8.4)	0.522
Mycophenolate mofetil	—	57 (44.5)	212 (39.5)	0.295
Cyclosporine	—	19 (14.8)	70 (13.0)	0.589
Tacrolimus	—	9 (7.0)	23 (4.3)	0.192
Azathioprine	—	8 (6.4)	42 (7.8)	0.545
Low-dose IL-2	—	16 (12.5)	203 (37.8)	<0.001***
SLEDAI-2k	≤ 4 points	54 (42.2)	377 (70.2)	<0.001***
	> 4 points	74 (57.8)	160 (29.8)	<0.001***

Data expressed as median (IQR) and n (%) and compared by Fisher’s exact test or Mann-Whitney *U*-tests.

***P<0.001

**P<0.01

*P<0.05.

IL-2, interleukin 2; Anti-dsDNA antibody, anti-double strand DNA antibody; SLEDAI-2k, Systemic lupus erythematosus disease activity index-2000.

We next performed propensity score matching, and 181 pairs of patients were selected from two groups, which enhanced the efficacy of statistical analysis by minimizing the effects of selection bias and controlling potential confounding factors (**S1 Table**). These matched patients from two groups had comparable characteristics except for the incidence of infections, with a lower incidence of infection in IL-2 group as compared to non-IL-2 group (8.3%, 15/181 v.s. 33.7%, 61/181, *P*-value < 0.001) (**Fig 1D**). Similarly, the multivariate analysis in matched groups showed that Ld-IL2 was a protective factor to reduce infection risk in SLE patients (OR = 0.13, 95% CI 0.07 to 0.23, *P*-value < 0.001) (**S1 Fig**). Together, despite the more severe disease and enhanced utility of corticosteroids in patients receiving Ld-IL2, this therapy indeed decreased the incidence of infection. It was suggested that Ld-IL2 is beneficial in preventing infections in patients with SLE.

### IL-2 treatment accelerates virus clearance and promotes effector CD8^+^ T cell response in influenza virus infection

**Fig 2:**
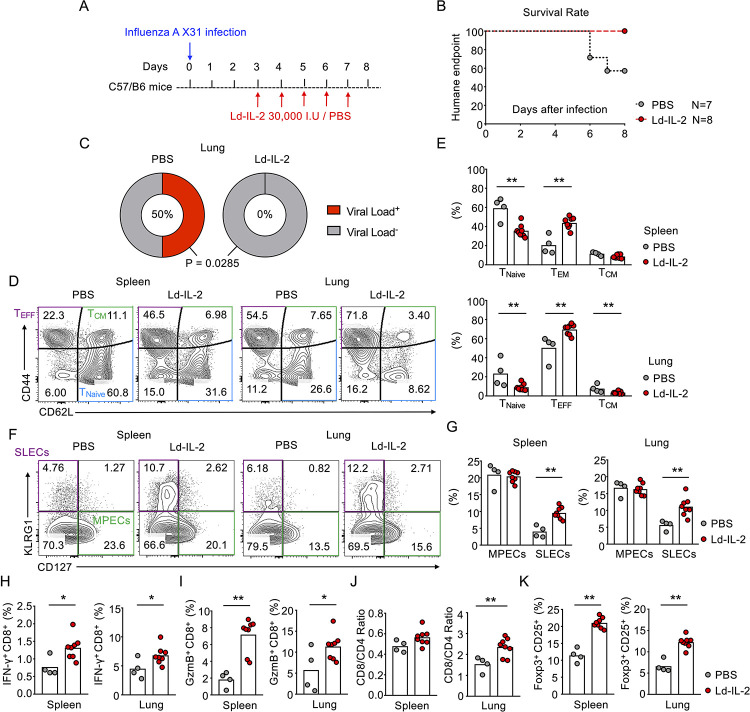
IL-2 treatment protects mice in influenza infection. (A-K) C57BL/6 mice were intranasally infected with 1x10^4^ PFU influenza A virus strain A/HKx31 (H3N2) and intraperitoneally treated with IL-2 (30,000 I.U) or PBS daily for 5 days from day 3 post infection. (A) Schematic for (B-K). (B) Humane endpoints of mice were recorded and compared between IL-2-treated and PBS treated mice were compared during LCMV infection (PBS, *n* = 7; IL-2, *n* = 8). (C) Percentage of mice with detectable virus load in lung tissue in PBS treated and IL-2-treated group. (D) CD8^+^ T cells were characterized as naïve cells (CD44^-^CD62L^+^), effector cells (CD44^+^CD62L^-^) and central memory cells (CD44^+^CD62L^+^), followed by the comparison of the frequency of each subset in spleen and lung between IL-2-treated and PBS treated mice 8 days post influenza infection, shown in FACs plots (D) and statistics (E). CD8^+^ T cells were also analyzed as short-lived effector cells (SLECs, KLRG1^+^CD127^-^) and memory precursor effector cells (MPECs, KLRG1^-^CD127^+^), and the frequency was compared in FACs plots (F) as well as statistics (G). IFN-γ and granzyme B producing CD8^+^ T cells in spleen and lung were analyzed in (H) and (I), respectively. (J) Ratios between CD8^+^ and CD4^+^ T cells in spleen and lung were calculated and compared between IL-2-treated and PBS treated mice. (K) Spleen and lung regulatory T cells (T_REG_, CD4^+^Foxp3^+^CD25^+^) were evaluated. Each dot represents one mouse with at least four mice per group and bars indicate mean values. Statistical significance was determined by Student t-test, or Chi-square test (c), *p < 0.05, **p < 0.01.

The association study of SLE patients suggested a beneficial role of Ld-IL2 therapy in controlling viral infection. Since upper respiratory tract infection was the most common form of infection in our human study (**Table 2**), we next evaluated the effect of Ld-IL2 therapy in a mouse model of influenza virus infection that also mimics local infection. Wide-type C57BL/6 mice were intranasally infected with the influenza A virus (X31). From day 3 post infection, mice were intraperitoneally injected with recombinant human IL-2 (30,000 international units (I.U.) daily) until day 7 (**Fig 2A**). This treatment regimen was widely used for Ld-IL2 therapy in mouse models [33,46]. We observed that IL-2 treatment significantly improved the health status with no mice reaching the humane endpoint (**Fig 2B**). At day 8 post infection, all mice in the IL-2 treated group (*n* = 8) had already cleared the virus in lung tissues while only 50% of the surviving mice in the PBS treated group had done so **(Fig 2C)**, indicating enhanced anti-viral immunity. CD8^+^ T cells are known to play a central role in controlling influenza infection [48]. Indeed, IL-2 treatment promoted the effector differentiation of CD8^+^ T cells, shown by a 2-fold expansion of CD44^+^CD62L^-^ effector population (T_EFF_) and 2-fold reduction in CD44^-^CD62L^+^ naïve population in splenic CD8^+^ T cells (**Fig 2D and 2E**). After influenza virus infection, CD8^+^ T_EFF_ cells particularly infiltrated the lung tissue, showing ~50% as compared to 20% in spleens. This was further enhanced to ~70% by IL-2 treatment (**Fig 2D and 2E**). KLRG1^+^CD127^-^ CD8^+^ T population represents short-lived and terminally differentiated effector cells (SLECs), which show robust cytolytic function in controlling infection and undergo rapid contraction after the resolution of infection [49]. IL-2 treatment increased the generation of SLECs in spleens and lung to about 2 folds, with negligible effect on the percentage of KLRG1^-^CD127^+^ memory precursor effector cells (MPECs) (**Fig 2F and 2G**). The promotion of CD8^+^ T cell cytolytic function by IL-2 treatment was also verified by the enhanced production of major cytotoxic molecules such as interferon gamma (IFN-γ) and granzyme B (**Figs 2H, 2I and S2A**). IL-2 treatment also enhanced the activation of CD4^+^ T cells (**S2B Fig**) but the effect was weaker compared to those in CD8^+^ T cells, demonstrated by a significant increase of CD8^+^/CD4^+^ ratio in lung tissues by IL-2 treatment (**Figs 2J and S2C**). As reported in other mouse models [33], IL-2 treatment expanded T_REG_ cells approximately 2-fold (**Fig 2K**). This evidence suggested that, despite the strengthened function of T_REG_ cells, IL-2 treatment promoted the generation of effector CD8^+^ T cells with enhanced cytotoxic functions, which underpinned better control of influenza virus infection. Such results echoed the observations in SLE patients and support the notion that IL-2 treatment can induce immune tolerance to treat autoimmune disease and might also enhance protective immunity to viral infection such as by influenza.

### IL-2 treatment accelerates virus clearance and promotes effector CD8^+^ T cell response in acute LCMV infection

**Fig 3:**
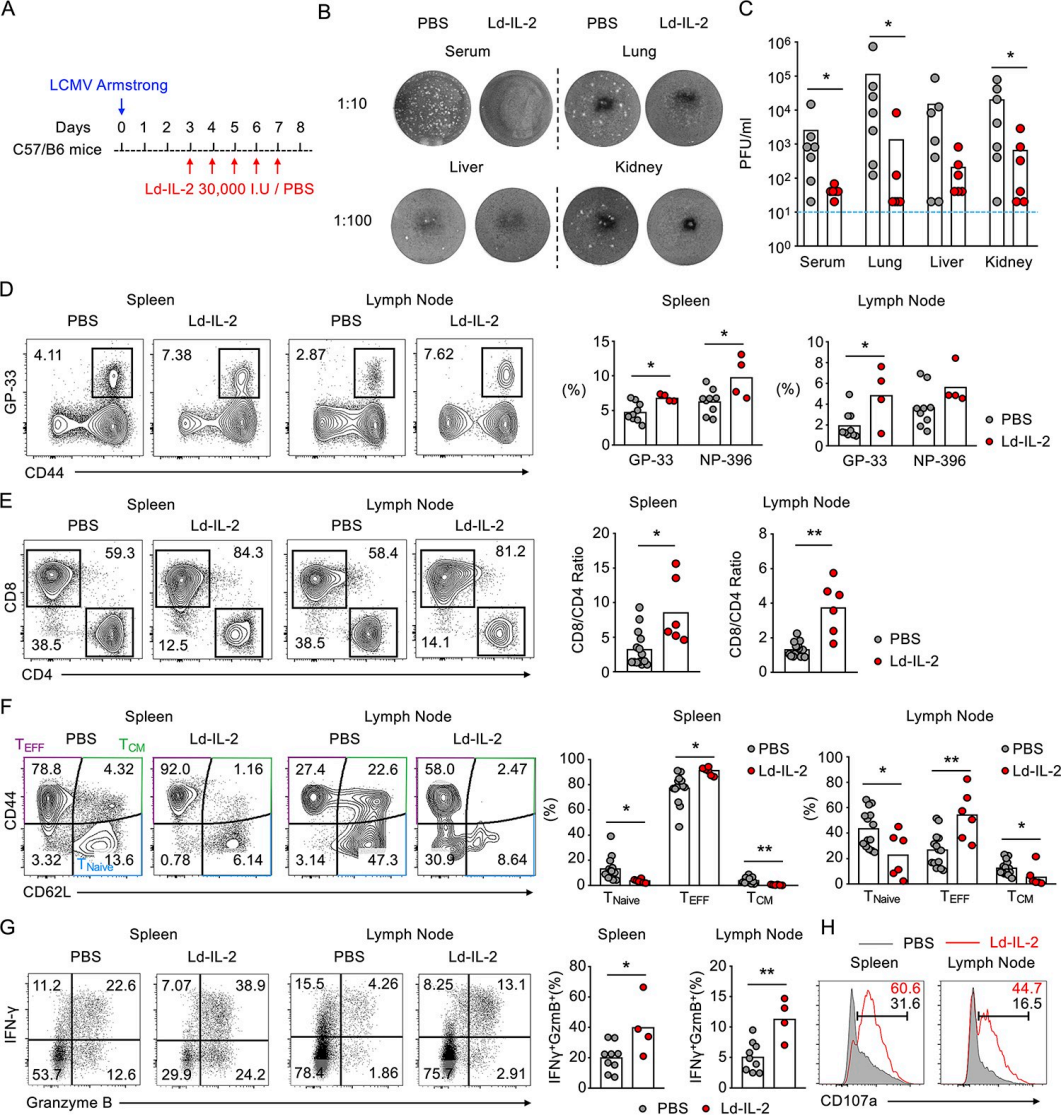
IL-2 treatment accelerates virus clearance and induces highly activated CD8^+^ T cells in acute LCMV infection. (A-H) C57BL/6 mice were intraperitoneally infected with lymphocytic choriomeningitis virus (LCMV) strain Armstrong (1x10^5^ PFU) and intraperitoneally treated with IL-2 (30,000 I.U) or PBS daily for 5 days from day 3 post infection. (A) Schematic for (B-H). (B) Plaque assays were performed on samples including serum, lung, liver and kidney collected on day 8 post infection, to demonstrate the changes of virus load between two groups of mice; Blue dash line indicates the undetectable value; Statistics were shown in (C). (D) Virus-specific CD8^+^ T cells were also analyzed using GP-33 and NP-396 tetramers, and the frequencies were compared between IL-2-treated and PBS treated mice. FACs plots (*left*) and statistics (*right*). (E) Frequency of CD8^+^ and CD4^+^ T cells were analyzed in flow cytometry (*left*) and ratios of CD8^+^ and CD4^+^ T cells in spleen and lymph nodes were calculated and compared between IL-2-treated and PBS treated mice (*right*). (F) CD8^+^ T cells were characterized as naïve cells (CD44^-^CD62L^+^), effector cells (CD44^+^CD62L^-^) and central memory cells (CD44^+^CD62L^+^), followed by the comparison of the frequency of each subset in spleen and lymph nodes between Ld-IL2 treated and PBS treated mice 8 days post infection. FACs plots (*left*) and statistics (*right*). (G) IFN-γ and granzyme B producing CD8^+^ T cells in spleen and lymph nodes were analyzed and shown in FACs plots (*left*) and statistics (*right*). (H) CD107a expression from CD8^+^ T cells were measured, and the representative plots were shown with the representative percentage. Each dot represents one individual mouse, and results are compiled from three independent experiments (E-F) or two independent experiments (D, G & H). Bars indicate mean values. Statistical significance was determined by Student t-test, *p < 0.05, **p < 0.01.

We next evaluated Ld-IL2 treatment in a systemic acute infection caused by lymphocytic choriomeningitis virus (LCMV) in mice. Mice were intraperitoneally infected with LCMV Armstrong and treated with the same regimen as in the influenza model (30,000 I.U. recombinant human IL-2 from day 3 to 7 post infection) (**Fig 3A**). Consistent with influenza infection, IL-2 treatment also promoted anti-viral immunity in acute LCMV infection, resulting in accelerated clearance of virus in serum and organs including lung, liver and kidney (**Fig 3B and 3C**). In the model of influenza infection, IL-2 treatment improved the viral control by enhancing effector differentiation and cytolytic function of CD8^+^ T cells (**Fig 2**). Similarly, IL-2 treatment in mice with acute LCMV infection also enhanced the generation of virus-specific CD8^+^ T cells recognizing dominant epitopes of glycoprotein (gp^33-41^) and, to a lesser extent, nucleoprotein (np^396-404^) (**Figs 3D, S3A and S3B**). Notably, compared to a modest increase in CD8^+^/CD4^+^ T cell ratio induced by IL-2 treatment in the model of influenza infection (**Fig 2J**), the same treatment in acute LCMV infection led to an approximately 3-fold increase in the CD8^+^/CD4^+^ T cell ratio in both spleen and inguinal lymph nodes (**Fig 3E**). Furthermore, IL-2 treatment almost completely diminished naïve and central memory CD8^+^ T cells while significantly expanding the effector population (**Fig 3F**). The polyfunctional effector CD8^+^ T cells expressing both IFN-γ and Granzyme B were doubled by IL-2 treatment (**Fig 3G**). Strikingly, the majority of CD8^+^ T cells in spleens and lymph nodes from IL-2-treated mice expressed the degranulation marker CD107α while a minority of CD8^+^ T cells in PBS-treated mice did so (**Fig 3H**). Collectively, these results further demonstrated that Ld-IL2 treatment markedly potentiate CD8^+^ T cells-mediated antiviral response.

### IL-2 treatment exacerbates LCMV induced-immunopathology in mice

**Fig 4:**
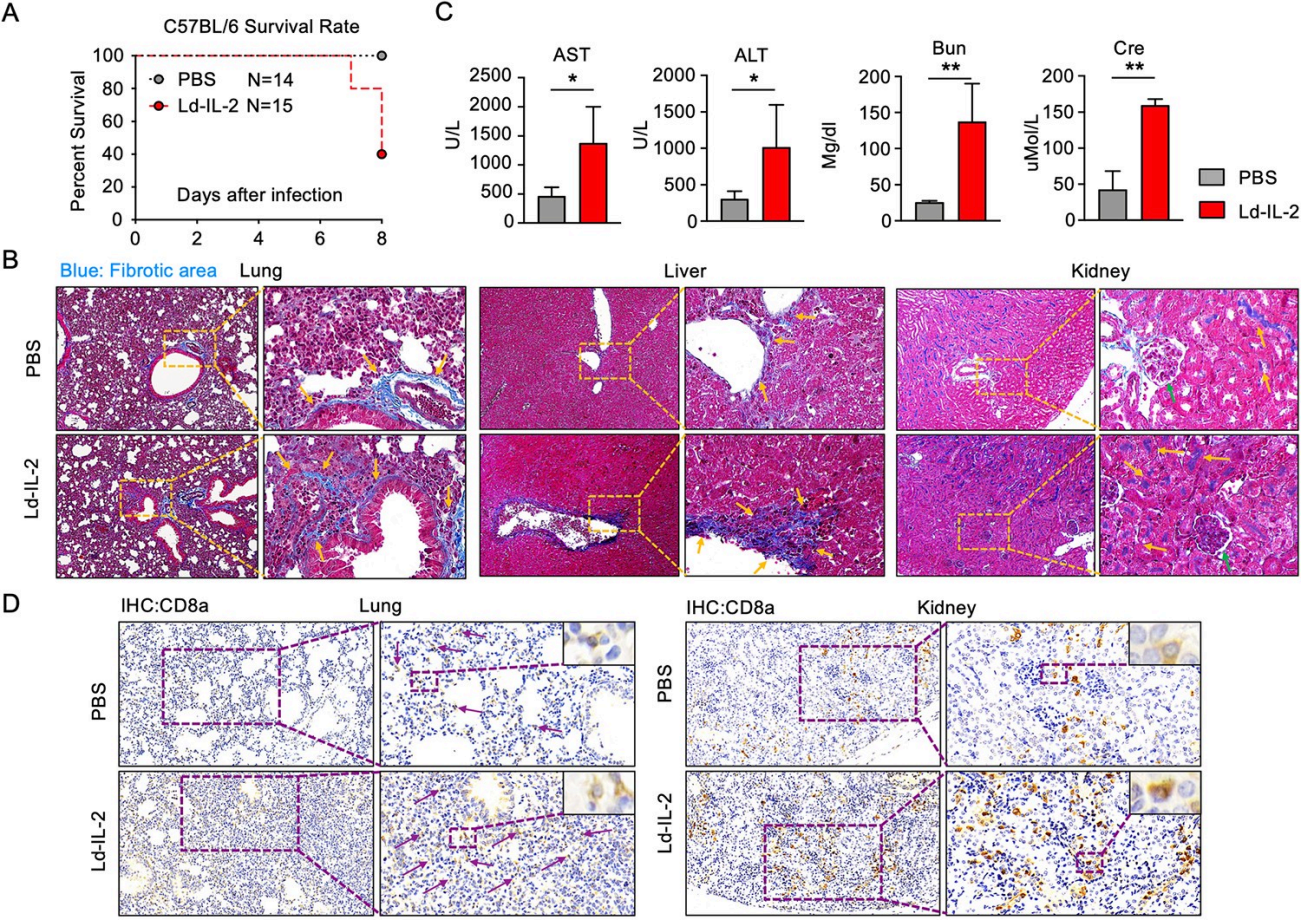
IL-2 treatment exacerbates immunopathology in acute LCMV infection. C57BL/6 mice (A-D) were intraperitoneally infected with lymphocytic choriomeningitis virus (LCMV) strain Armstrong (1x10^5^ PFU) and intraperitoneally treated with IL-2 (30,000 I.U) or PBS daily for 5 days from day 3 post infection. (A) Mortality of mice was recorded and survival rates between IL-2 treated and PBS treated mice were compared during LCMV infection (PBS, *n* = 14; IL-2, *n* = 15). (B) Masson’s trichrome staining was performed to show the fibrotic injuries in lung, liver and kidney on day 8 post LCMV infection. Magnifications were 100x for images in the left which were enlarged to 400x in the right. Arrows were indicating the fibrotic injuries. (C) Aspartate aminotransferase (AST) and alanine aminotransferase (ALT) were measured to evaluate liver damage, and blood urea nitrogen (Bun) and creatinine in serum were measured to evaluate kidney damage (PBS, *n* = 5; IL-2, *n* = 5). (D) Immunohistochemistry was performed to stain CD8^+^ T cells in lung (*left*) and kidney (*right*) using anti-CD8a antibody 8 days post infection. Magnifications are 200x for images in the left and 400x in the right. Arrows indicate CD8^+^ T cells. Three independent experiments were performed with at least four mice per group and bars indicate mean values. Statistical significance was determined by Student t-test, *p < 0.05, **p < 0.01.

Noticeably, despite better control of virus, IL-2 treatment in mice infected with acute LCMV led to 60% of mice reaching humane endpoint compared to 0% in the PBS treated control group (**Fig 4A**). Due to the decreased virus load in IL-2 treatment group (**Fig 3C**), the deterioration by IL-2 treatment was unlikely caused by cytopathic effects of virus. LCMV infection has been reported to induce significant immunopathology [50–52]. Therefore, we examined the pathology in organs of infected mice. Marked increase in immune cell infiltration, more necrosis and worse tissue integrity were found in mice treated with IL-2 compared to control mice treated with PBS **(S3C Fig)**. The induction of more severe damage to multiple tissues by IL-2 treatment was further demonstrated by exacerbated fibrosis in lung, liver, and kidney (**Figs 4B and S4**) and elevated serum levels of aspartate aminotransferase (AST), alanine aminotransferase (ALT), urea nitrogen and creatinine (**Fig 4C**). These results revealed that, despite better control of LCMV infection, IL-2 treatment caused severe illness in mice, which was associated with exacerbated immunopathology in multiple tissues. Compared to the moderate enhancement of CD8^+^ T cell activation in influenza infection, IL-2 treatment in acute LCMV infection caused a stronger activation of CD8^+^ T cells (**Fig 3E–3H**), which might break down the immune tolerance and result in CD8^+^ T cell-mediated tissue immunopathology. Immunohistochemical analysis demonstrated that IL-2 treatment induced a vast infiltration of CD8^+^ T cells in lung and kidney (**Figs 4D and S4**). The accumulation of CD8^+^ T cells surrounding the glomerulus in kidney in the IL-2-treated mice might lead to disrupted glomerulus integrity and impaired kidney function (**Fig 4D**), causing the 4-6-fold increase in serum urea nitrogen and creatinine (**Fig 4C**).

### CD8^+^ T cells mediate immunopathology in LCMV infected mice treated with IL-2

**Fig 5:**
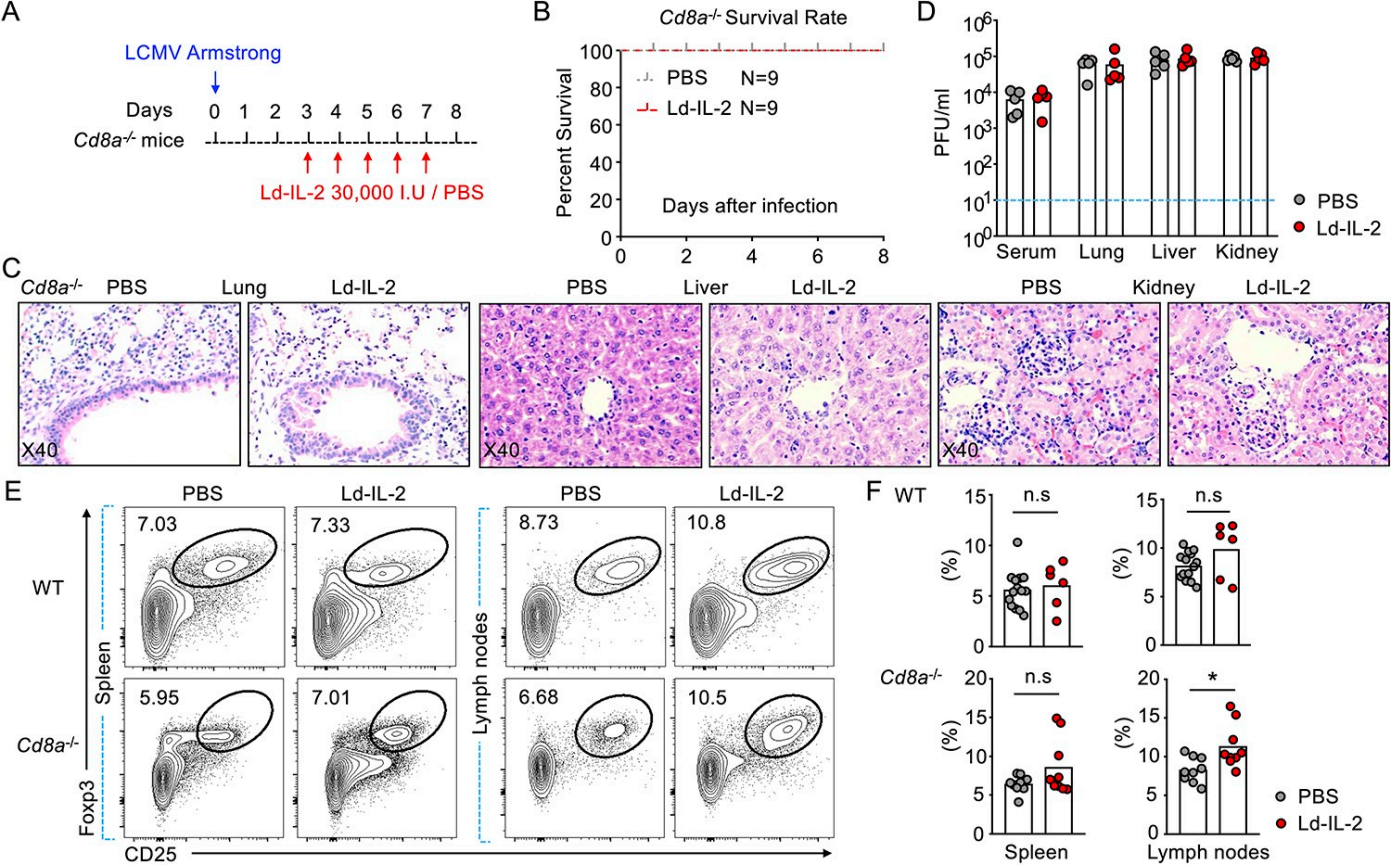
CD8^+^ T cells mediate IL-2-deteriorated immunopathology after IL-2 treatment. *Cd8a*^-/-^ mice were intraperitoneally infected with lymphocytic choriomeningitis virus (LCMV) strain Armstrong (1x10^5^ PFU) and intraperitoneally treated with IL-2 (30,000 I.U) or PBS daily for 5 days from day 3 post infection. (A) Schematic for (A-F). (B) Mortality of mice was recorded and survival rates between IL-2-treated and PBS treated mice were compared during LCMV infection (PBS, *n* = 9; IL-2, *n* = 9). (C) Hematoxylin & Eosin staining was performed to show the pathology in lung, liver and kidney on day 8 post LCMV infection. Magnifications were 400X in each image. (D) Plaque assay was used to demonstrate the virus load from serum, lung, liver and kidney on day 8 post infection shown in statistics representing two independent experiments. (E) Regulatory T cells (T_REG_, CD4^+^Foxp3^+^CD25^+^) in spleen and lymph nodes were evaluated in wild-type mice (*up*) and *Cd8a*^-/-^ mice (*bottom*) 8 days post LCMV infection, treated with or without IL-2, and statistics in (F). Each dot represents one individual mouse, and results are compiled from three independent experiments (E, *up*) or two independent experiments (E, *bottom*) with at least four mice per group and bars indicate mean values. Statistical significance was determined by Student t-test, *p < 0.05, **p < 0.01.

To further understand the mechanisms underlying the deteriorated immunopathology in mouse LCMV infection, we examined the effect of IL-2 treatment in *Cd8a*^-/-^ mice with no CD8^+^ T cells. In the same manner as wildtype mice (**Fig 3A**), *Cd8a*^-/-^ mice were infected with LCMV Armstrong and treated with recombinant human IL-2 (**Fig 5A**). Both groups showed no signs of severe illness and thus survived over the course of the experiment (**Fig 5B**), in contrast to around 60% of IL-2-treated wildtype mice reaching humane endpoints (**Fig 4A**). Analyzing tissues histologically showed comparable cell infiltration and tissue morphology in *Cd8a*^-/-^ mice with or without IL-2 treatment (**Fig 5C**). No exacerbation of illness was observed in IL-2-treated *Cd8a*^-/-^ mice suggesting that CD8^+^ T cells mediate severe immunopathology and illness. While the lack of CD8^+^ T cells limited IL-2 treatment-induced immunopathology, IL-2 no longer enhanced the clearance of virus in the absence of CD8^+^ T cells **(Fig 5D)**.

T_REG_ cells are important in limiting immunopathology and to resolve the inflammation caused by anti-viral immunity during viral infection [53,54]. IL-2 treatment expanded Foxp3^+^CD25^+^ T_REG_ cells about 2-fold in mice infected with influenza virus (**Fig 2K**). However, the same treatment failed to expand Foxp3^+^CD25^+^ T_REG_ cells in acute LCMV infection (**Fig 5E and 5F**). It has been reported that T_REG_ cells can compete for IL-2 to restrain the function of other immune cells including CD8^+^ T cells and NK cells [55,56]. We therefore speculated that the hyper-activation of CD8^+^ T cells in acute LCMV infection might sequester IL-2 from T_REG_ cells. We then examined *Cd8a*^-/-^ mice infected with LCMV and found that the expansion of T_REG_ cells by IL-2 treatment was partially restored, showing a statistically significant increase in lymph nodes but the increasing trend in spleens didn’t reach statistical significance (**Fig 5E and 5F**). Therefore, the strong activation of CD8^+^ T cells in systemic infection such as LCMV infection outcompeted T_REG_ cells in accessing treated IL-2, which diminished the expansion of T_REG_ cells and abolished the anti-inflammatory benefit of Ld-IL2 therapy.

## Discussion

By inducing immune tolerance [30], Ld-IL2 therapy is emerging as a new approach to treat autoimmune and inflammatory diseases and has shown safety and promising efficacy in a broad range of conditions including GVHD [38], SLE [37,46,47,57,58], type 1 diabetes [41,42,59] and hepatitis C virus (HCV) induced vasculitis [40]. Compared to conventional immunosuppressive treatments including glucocorticoids, Ld-IL2 therapy potentially possesses a highly sought-after advantage–reinstate immune tolerance without imposing immunosuppression, therefore alleviating the significant risk of infection in patients with autoimmune diseases. The very early study suggested that a single shot of Ld-IL2 enabled uraemic patients to respond to the vaccination for hepatitis B virus [60]. More recently, Ld-IL2 was shown to expand T_REG_ cells to improve autoimmune condition in patients with HCV-induced vasculitis, without suppressing anti-viral immunity and increasing HCV viral loads [40]. In the recent double-blind clinical trial of Ld-IL2 therapy in SLE patients, we recorded a trend of a lower incidence of infection in the IL-2 group (6.9%, 2/29) compared with the placebo group (20.0%, 6/30) but the result did not reach a statistical significance [47]. Therefore, despite several lines of evidence for Ld-IL2 therapy in maintaining the immunity to infection, a formal evaluation of how Ld-IL2 therapy might impact infections in autoimmune and inflammatory diseases is still lacking.

**Fig 6:**
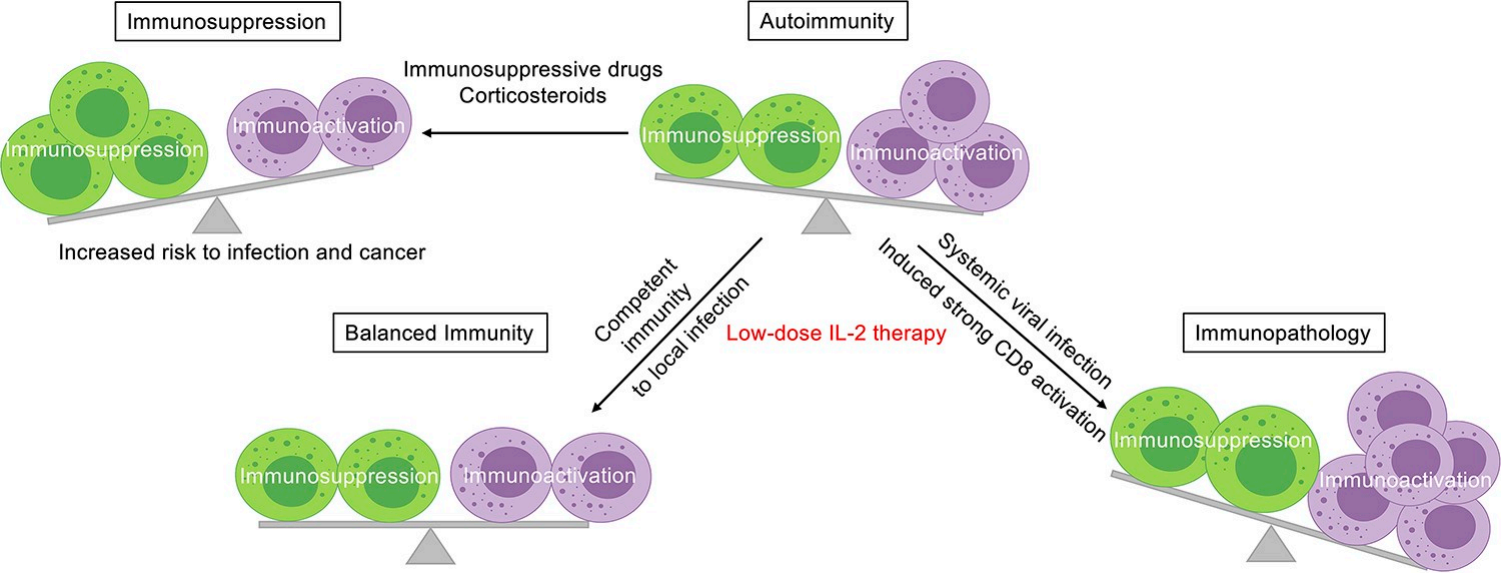
Schematics of immunosuppression and immunoactivation balanced by Ld-IL2 treatment in autoimmunity. Our running model of Ld-IL2 therapy in the context of immunoactivation and immunosuppression in autoimmunity. Comparing to corticosteroids and other immunosuppressive medications, Ld-IL2 therapy can induce competent immunity towards infection when treating autoimmunity. However, this balance has to be cautiously monitored as IL-2 treatment might exacerbate immunopathology mediated by strongly activated CD8^+^ T cells in mice. Illustration was created with BioRender.com.

Therefore, we first conducted this observational study of a sizable cohort of SLE patients including over 200 cases treated by Ld-IL2 in addition to standard therapy. In a six-month following up, we recorded a significant burden of infections in the group of conventional therapies that affecting 25.1% of patients. The incidence rate of infection was largely consistent with other studies which varied from 26% to 78% with follow-up time generally over one year [17,25,61–63]. According to the consensus recommendation, Ld-IL2 therapy should be prescribed to SLE patients with disease flares [64]. In agreement with this, patients in the IL-2 group showed higher disease activity scores and higher doses of corticosteroids than those in the non-IL-2 group. Despite the positive association between infection risk and disease activity and corticosteroid dose [24], we observed a 17.8% (7.3% in the IL-2 group *v*.*s* 25.1% in non-IL-2 group) decrease of total infection incidence in the IL-2 group, especially in herpes and upper respiratory tract infection. These results represent the first evidence, based on published data, supporting the notion that Ld-IL2 therapy in autoimmune diseases can benefit patients by reducing the risk of infection, a feature outcompeting conventional immunosuppressive therapy (**Fig 6**).

We further validated this observation using mouse models on infection and found that IL-2 treatment largely improved illness in mice infected with influenza A virus with no mice reaching the humane endpoints, compared to about 40% of PBS treated mice reaching humane endpoints **(Fig 2B)**. Other studies also demonstrated the benefit of IL-2 in controlling influenza infection, shown by the protection in mice infected with influenza A virus engineered to express IL-2, which improved CD8^+^ T cell immunogenicity in the mouse model [65]. In our experiment, mice receiving IL-2 treatment showed an increased frequency of effector CD8^+^ T cells, characterized by both effector memory (CD44^+^CD62L^-^) as well as short-lived effector (KLRG1^+^CD127^-^) phenotypes in influenza infection, together with the increased production of granzymes and IFN-γ **(Fig 2D, 2F, 2H and 2I)**. These suggest that IL-2-enhanced the generation of effector CD8^+^ T cell response that controlled viral infection in mice, providing a rationale of why Ld-IL2 treatment sustains anti-viral immunity in SLE patients.

Though less common, certain infections in autoimmune patients could develop into systemic infections and induce more severe clinical symptoms. Moreover, severe infections could induce systemic damage in multiple organs, including acute respiratory distress syndrome (ARDS) and acute renal failure [66,67]. Would Ld-IL2 therapy still be beneficial? To answer the question, we adopted the mouse LCMV infection as the model for systemic immune activation. While IL-2 treatment markedly reduced virus loads, such improved clearance of pathogens was accompanied by ~60% death in IL-2-treated mice comparing to control mice **(Fig 4A)**. LCMV is known to induce immunopathology in organs such as lung and liver [52]. We further confirmed that IL-2 treatment deteriorated the immunopathology in lung, liver, and kidney, evidenced by increased cellularity, fibrosis, and severe injuries **(Fig 4B)**. In the LCMV model, we noticed that IL-2 treatment increased the ratio of CD8^+^ T cells to CD4^+^ T cells and increased the infiltration of CD8^+^ T cells into the lung and kidney **(Fig 4D)**. It has been shown that activated CD8^+^ T cells instigate cachexia during chronic LCMV infection [68]. Studies have also suggested that strongly activated CD8^+^ T cells are responsible for immunopathology in organs such as brain, lung, liver, and skin during infection [69–72]. We also found that IL-2-deteriorated immunopathology after IL-2 treatment largely resulted from an increased accumulation of activated tissue infiltrating CD8^+^ T cells, which was markedly rescued in *Cd8a* knock-out mice (**Figs 5 and 6**).

There is an equilibrium of T_REG_ cells and CD8^+^ T cells to compete for IL-2 in infection [73]. In influenza infection in mice, IL-2 treatment competently induced the expansion of T_REG_ cells besides the activation of CD8^+^ T cells **(Fig 2K)**. However, IL-2-induced expansion of T_REG_ cells was largely diminished in mice infected by LCMV **(Fig 5E and 5F)**. In systemic viral infection such as the LCMV model, overwhelmingly activated CD8^+^ T cells may sequester IL-2 from T_REG_ cells. In line with this hypothesis, in *Cd8a*^-/-^ mice, the expansion of T_REG_ cells by Ld-IL2 was partially restored **(Fig 5E and 5F)**.

Significantly activated CD8^+^ T cells can lead to hemophagocytic lymphohistiocytosis (HLH), an autoimmune and life-threatening disease with severe systemic immunopathology and often associates with infection and cancer [74,75]. CD8^+^ T cells with high IFN-γ production are one of the major contributors to this disease [76]. Neutralization of IFN-γ using monoclonal antibody alleviated HLH in mouse model [77]. As our model displays the similar disease features, it is thus interesting in future studies to test whether combination therapy of IL-2 with anti-IFN-γ monoclonal antibody could solve the immunopathology without impairing the competent antiviral immunity by CD8^+^ T cells.

Although the cohort study of SLE patients and mouse influenza models suggest a significant benefit of Ld-IL2 therapy for infection control, the revelation of deteriorated inflammation and organ damages by IL-2 treatment in the mouse model of LCMV infection represents an important alarming issue for patients with hyper-reactive CD8^+^ T cell immune response. We would like to clarify that although there could be some risk, we did not record the immunopathology in our cohort of SLE patients received Ld-IL2 therapy. Nevertheless, to closely monitor the potential severe complication in the Ld-IL2 therapy, it could be useful to examine the patients’ peripheral CD4^+^ and CD8^+^ T cell ratio as markedly increased CD8/CD4 ratio might indicate the hyperactivation of CD8^+^ T cells by Ld-IL2 treatment which might lead to severe immunopathology as we have found in mouse LCMV infection. Ld-IL2 therapy was registered for clinical trials on COVID-19 patients *(ClinicalTrials*.*gov)* [78,79]. Recent studies reported that, in many COVID-19 patients, CD8^+^ T cells presented the hyperactivation feature, including high levels of markers for cytotoxicity, increased numbers of CD38^+^HLA-DR^+^ activated population, and more Ki67^+^ proliferating cells [80–83]. Interestingly, we also identified that soluble CD25 (sCD25), which constitute the IL-2 receptor subunit, suggests a divergence between anti-viral and pro-inflammatory T cell responses in severe COVID-19 patients [84]. Therefore, the results of Ld-IL2 therapy in COVID-19 patients will be informative and helpful to understand the multiple roles of Ld-IL2 therapy in controlling infection, expanding T_REG_ cells [79], regulating CD4^+^ T cell subsets [82,85], enhancing CD8^+^ T cells activation or exhaustion [83] or potentially inducing bystander CD8^+^ T cell-mediated immunopathology [86] in COVID-19 and other disease.

## Conclusion

In summary, the investigation of Ld-IL2 therapy in patients and mouse models provided insights for the clinical application and further optimization of Ld-IL2 therapy for autoimmune patients, particularly for those with the risk of infections.

## Materials and methods

### Ethics statement

All human studies were conducted in line with the ethics protocols approved by Peking University People’s Hospital and verbal consent was obtained from the participants. All animal experiments were performed under the guidelines approved by the Animal Ethics Committees of The Australian National University. This study was approved by Peking University People’s Hospital Ethics Committee and the Animal Ethics Committees of The Australian National University.

### Study design

A retrospective cohort study was performed to evaluate the infection and relative risks using data for 665 systemic lupus erythematosus (SLE) patients ages 18–75 years enrolled in the Department of Rheumatology and Immunology at Peking University People’s Hospital from 2016 to 2018. Multivariate binary logistic regression analyses were performed to investigate the association between baseline variables and infection. To further validate the clinical observations, we analyzed samples from age and gender matched mice infected by influenza A virus (IVA) and lymphocytic choriomeningitis virus (LCMV) respectively. In general, these infected mice were also treated with low-dose IL-2 (Ld-IL2) (30,000 I.U) for 5 consecutive days from day 3 post infection then sacrificed for examination. Evaluations from infected mice include but not limit to survival rate, body weight, virus titer, anti-viral immune response, and pathology. Both human and mouse studies were in line with the ethics protocols approved by Peking University People’s Hospital and The Australian National University.

### Patients

We reviewed the SLE patients who regularly visited the Department of Rheumatology and Immunology at Peking University People’s Hospital, Beijing, China from December 2016 to August 2018. There were in total 665 SLE patients included with this study, who had detailed medical records and were fulfilled the 1997 revised criteria of the American College of Rheumatology (ACR). Of these patients, 219 were treated with “conventional agents and Ld-IL2 (IL-2 cohort)”, and 446 were treated with “conventional agents alone (non-IL-2 cohort)”. Conventional agents were any dose with corticosteroid, antimalarial and immunosuppressive agents. Ld-IL2 (1 million international unites, I.U) was administered subcutaneously every other day for 2 weeks and followed by a 2-week break as one treatment cycle (4-week), with a total of 3 treatment cycles (12-week) and a 12-week follow-up. The date where SLE patients started receiving Ld-IL2 therapy was defined as Day-1. Correspondingly, matched patients in the non-IL-2 group were recruited in our study with the matched parameters including the same Day-1, age (± 1 year) and sex. The infection episodes and clinical data were recorded during the 6-month treatment period through a comprehensible pre-established questionnaire, including medical history and examination for disease and infection, by the rheumatologists in charge of the patients at their visits. The enrolment of this study began in December 2016 and ended in February 2018, 6 months before the closure of our study in August 2018. Verbal consent was obtained from the participants. This study was approved by Peking University People’s Hospital Ethics Committee.

### Definition of infection in SLE patients

Infections were confirmed at least by one infectious disease specialist or rheumatologist during an infection episode. Measurements include the evaluation of clinical symptoms, positive microorganism culture and response to antibiotic therapy. Bacterial and fungal infections were defined as clinical symptoms and/or signs of infection with an organism isolated from the site of infection or blood culture in combination with the antibiotic therapy response. Viral infections, mainly upper respiratory tract infection, were confirmed when patients had acute pharyngitis, rhinitis, or other typical symptoms. Other viral infections, including cytomegalovirus, herpes zoster, Epstein-Barr virus, were determined by clinical manifestations, laboratory abnormalities, and detection of DNA or RNA or antibodies for specific antigen.

### Clinical analysis of patient data

Statistical analyses for baseline demographic and clinical characteristics were described all the variables, including frequency and percentage for categorical variables, mean (SD) and median (interquartile ranges) for normal or abnormal distribution continuous variables. The statistical significances between groups were assessed using the Student’s t-tests, Mann-Whitney U-tests and Chi-square (χ^2^) test. Logistic regression analysis was performed to identify the risk factors of infection. Statistical analyses were performed using SPSS for Mac version 22.0 (IBM, Corp., Armonk, NY, USA).

To reduce the influence of treatment selection bias in this study, we performed a one-to-one propensity score-matching analysis between the IL-2 and non-IL-2 cohort based on the estimated propensity scores (PS). PS is calculated using a logistic regression model based on the following factors, including gender, age, duration, systemic lupus erythematosus disease activity index-2000 (SLEDAI-2k), medications. The c-statistic was 0.02 for assessing the efficacy of fit and sensitivity analyses were performed after the PSM analysis. Statistical analyses were performed using SPSS.

### Mice

6-to 8-week C57BL/6 female mice were used in this study. *Cd8a*-deficient (CD8a^-/-^) mice were sourced from Australian Phenomics Facility. All experimental mice were maintained in a specific pathogen-free facility at the Australian Phenomics Facility of The Australian National University, Canberra. Age and sex-matched mice were utilized for experiments. All procedures were approved by the Animal Ethics Committees of Australian National University.

### Viral infection

Influenza A virus strain A/HKx31 (H3N2) was provided by Prof. Katherine Kedzierska from University of Melbourne. To induce primary anti-viral immune responses, 6 to 8-week-old Charles River C57BL/6 mice were anesthetized by inhalation of isoflurane prior to being intranasally infected with 1 × 10^4^ PFU (plaque-forming units) of A/HKx31 influenza A virus in 30 μl of PBS. 30,000 I.U (international unites) of IL-2 were intraperitoneal injected (i.p) into mice daily for 5 times 3 days post infection. Mice were sacrificed on day 8 post infection and samples were collected for further examination. To study the role of Ld-IL2 in acute lymphocytic choriomeningitis virus (LCMV) infection, C57BL/6 mice and *Cd8a*-deficient (CD8a^-/-^) mice were intraperitoneally (i.p) infected with 1 x 10^5^ LCMV Armstrong virus. LCMV Armstrong virus was provided by Prof. Scott N Muller from University of Melbourne, then propagated and collected using BHK cell line which was cultured with complete DMEM medium. 30,000 I.U (international units) of IL-2 were intraperitoneal injected (i.p) into mice daily for 5 times 3 days post infection. Mice were sacrificed on day 8 post infection and samples were collected for further examination. All infected mice were closely monitored and scored according to the mouse infection scoring index in **S2 Table** and ANU SOP "Mouse Clinical Monitoring Card” to determine the humane endpoints.

### Quantification of virus load

Tissue homogenates and serum from LCMV or influenza infected mice or were titered on Vero E6 cells. Briefly, ten-fold serial dilutions of tissue homogenates and serum were co-cultured with Vero E6 cells. After 60 minutes of incubation at 37°C, 5% CO2, complete DMEM medium containing 0.3% carboxymethylcellulose (CMC) supplemented with 5% heat inactivated fetal bovine serum, penicillin and streptomycin was added. After 5 days, cells were fixed in 1% formaldehyde in phosphate buffered saline (1%PFA/PBS) for 1 hour or overnight and stained with 0.1% toluidine blue solution to determine plaque numbers. Plaques were manually counted using a light microscope. Virus titer was calculated by multiplying the number of plaques by the dilution factor and ratio of volume plated in 1 mL per sample to determine virus titer per tissue sample per mouse. Formulae (no of plaques *dilution factor)/ (1 mL/volume of inoculum plated).

### Flow cytometry

Fc-receptor blocking antibodies (clone 2.4G2, 1:100 dilution, BD) were used to block non-specific staining on mouse splenocytes or lymphocytes for 15 min on ice. For surface staining, cells were washed once with FACs buffer which was PBS containing 2% heat-inactivated fetal bovine serum (FBS, Gibco) and incubated with appropriately diluted primary antibodies in FACs buffer for 1 hour at 4°C followed by 30 min of streptavidin (BD) staining at same temperature if needed. To detect LCMV antigen-specific CD8^+^ T cells, APC-conjugated H-2Db-GP33-tetramer or APC-conjugated H-2Db-NP396-tetramer were stained with primary antibodies. These tetramers were sourced from Department of Immunology and Microbiology, University of Melbourne or manufactured using peptides by Biomolecular Resource Facility (BRF) at John Curtin School of Medical Research, The Australian National University. GP33 peptide sequence was KAVYNFATC and NP396 peptide sequence was FQPQNGQFI. The 7-amino-actinomycin D (7-AAD, Thermo Fisher) was stained to exclude dead cells.

For intracellular staining, cells were washed once after surface staining and permeabilized using Cytofix/Cytoperm (BD) for 40 min on ice. Antibodies were then diluted in Perm/Wash Buffer (BD) and stained for intracellular antigens at appropriate concentrations for 1 hour at 4°C. For intranuclear staining, cells were washed once after surface staining and permeabilized using Foxp3/Transcription Factor Staining Buffer Set (eBioscience) for 40 min on ice. The specific antibodies were diluted in Fixation/permeabilization buffer (eBioscience) and incubated for 60 min at 4°C. For studying mouse CD8^+^ T cells or CD4^+^ T cells, lymphocytes were gated on 7AAD^-^B220^-^CD3^+^CD8^+^ or B220^-^CD3^+^CD4^+^, respectively. Data were collected on a BD LSR Fortessa (BD) and analyzed using FlowJo software. Antibody information is presented in **S3 Table**.

### Tissue histology

To evaluate immunohistology in organs, mice were ethically sacrificed at end-point day and lung, kidney, liver tissues were collected for histology staining. All samples were fully fixed with 4% paraformaldehyde, paraffin-embedded, and cut into 5-μm sections, then stained with Hematoxylin & Eosin. Sections were permeabilized with xylene and mounted with neutral balsam. Images were collected with microscope (NIKON ECLIPSE CI) and morphological evaluations were determined at the magnification of 50X or 400X in a series of randomly selected tissue sites from lung, liver, and kidney.

To evaluate the fibrotic injury in different organs, we performed Masson’s trichrome staining on tissue sections mentioned above. The paraffin embedded sections were incubated in potassium dichromate overnight then stained with Masson’s trichrome staining kit (Servicebio G1006). Specifically, sections were stained with iron hematoxylin staining solution for 3 min. Next, sections were immersed in ponceau staining solution for 5–10 min after being fully washed with running warm tap water and distilled water, respectively. Following this, sections were stained with phosphomolybdic acid for 1–3 min then directly incubated with aniline blue for 5 min before being stained with 1% acetic acid aqueous solution for 1 min. Slices were cleaned with xylene and mounted with neutral balsam. Images were collected with microscope (NIKON ECLIPSE CI) at the magnification of 100X or 400X in a series of randomly selected tissue sites from lung, liver, and kidney. Immunopathology was scored blindly with method described previously [87].

### Immunohistochemistry

To measure the infiltration of CD8^+^ T cells in organs, immunohistochemistry staining was conducted on lung, kidney and liver tissues. Tissue samples were fully fixed with 4% paraformaldehyde, paraffin-embedded, and cut into 5-μm sections. Heat-induced antigen retrieval in citrate buffer (10 mM citric acid, 0.05% Tween 20, pH 6.0) for 25 min at 95 to 100°C was used, and endogenous peroxidase was blocked with 3% H_2_O_2_ for 25 min, followed by incubation with 3% normal bovine serum for another 30 min. Primary antibody against CD8a (GB11068, 1:1500, Servicebio) was incubated overnight at 4°C, followed by detection with HRP (horseradish peroxidase) conjugate (goat anti rabbit antibody, Servicebio, G23303, 1:200) then developed with DAB Chromogen. Next, sections were counterstained with haematoxylin for 3 min and washed with water. Slides were mounted with neutral balsam and scanned with Pannoramic DESK (3D HISTECH, Hungary). Randomly selected areas were analyzed with Pannoramic Viewer (P.V 1.15.3) at the magnification of 200X or 400X.

### Biochemistry analysis

To validate the organ injuries, biochemistry analysis was performed to measure the blood level of key enzymes for kidney and liver injury. Blood was collected from LCMV infected mice on end-point day and serum was separated using centrifuge (12000 RPM, 20 min, room temperature). Collected serum was diluted at 1:4 and used for further biochemistry analysis. To evaluate liver damage, aspartate aminotransferase (AST) and alanine aminotransferase (ALT) were measured by ALT and AST Activity Assay Kit (Changchun huili). To evaluate kidney damage, blood urea nitrogen (Bun) and creatinine in serum were measured by Urea Nitrogen Detection Kit and Creatinine Assay Kit (Changchun huili). The readouts were generated and analyzed by Automatic Biochemistry Analyzer Chemray 240 (Rayto Life and Analytical Science, Shenzhen, China) according to the manufacturer’s instructions.

### Statistical analysis

Methods used for statistical analysis of clinical data have been described above. All mouse experimental data were analyzed by unpaired Student t-test or Chi-square test using GraphPad Prism 8.0 software. All values from mouse experiment data were expressed as mean and bar graph indicates the mean value. Clinical data were analyzed by Mann-Whitney U-test and Fisher’s exact test using SPSS 22.0 IBM. Differences were considered to be statistically different at *p < 0.05, **p <0.01, *** p < 0.001.

## Supporting information

S1 Table Characteristics of SLE patients after propensity score-matching. (DOCX)

S2 Table Mouse infection scoring index. (DOCX)

S3 Table Key Resources Table. (DOCX)

S1 Fig Risk factors of infection with multivariate analysis after propensity score matching in SLE patients. Low-dose IL-2 was a protective factor of infections in SLE patients (P < 0.001, odd ratio 0.13, 95% CI [0.07 to 0.23]). Disease duration, lymphopenia, hypocomplementemia, dsDNA> 25 IU/mL, SLEDAI > 4, Pred > 30 mg/d were risk factors of infection in SLE patients (P < 0.05). Data were analyzed by binary logistic regression test. dsDNA, anti- double strand DNA antibody; Pred, Prednisolone; IL-2, interleukin 2; SLEDAI-2k, Systemic lupus erythematosus disease activity index-2000.
(TIF)

S2 Fig Low-dose IL-2 therapy protects the mice in influenza infection. (a-c) C57BL/6 mice were intranasally infected with 1x10^4^ PFU influenza A virus strain A/HKx31 (H3N2) and intraperitoneally treated with low-dose IL-2 (30,000 I.U) or PBS daily for 5 days from day 3 post infection. (a) IFN-γ and granzyme B producing CD8^+^ T cells in spleen, lung and lymph nodes were shown in FACs plots. (b) CD4^+^ T cells were characterized as naïve (CD44^-^CD62L^+^), effector memory (CD44^+^CD62L^-^) and central memory (CD44^+^CD62L^+^) type of cells, followed by the comparison of the frequency of each subset in spleen and lung between low-dose IL-2 treated and PBS treated mice 8 days post influenza infection. (c) Cell numbers of CD8+ and CD4+ T cells in spleen and lung were calculated and compared between low-dose IL-2 treated and PBS treated mice. Each dot represents one mouse from two independent experiments with at least four mice per group and bars indicate mean values. Statistical significance was determined by Student t-test, *p <0.05, **p <0.01.
(TIF)

S3 Fig Low-dose IL-2 therapy promotes NP-396 specific CD8^+^ T cells while increases cellularity and damages tissue integrity in organs during LCMV infection. (a-c) C57BL/6 mice were intraperitoneally infected with lymphocytic choriomeningitis virus (LCMV) strain Armstrong (1x10^5^ PFU) and intraperitoneally treated with low-dose IL-2 (30,000 I.U) or PBS daily for 5 days from day 3 post infection. (a) NP-396 virus- specific CD8^+^ T cells were analyzed tetramer, and the frequency was compared between low-dose IL-2 treated and PBS treated mice shown in FACs plots. (b) Cell numbers of CD8^+^ and CD4^+^ T cells in spleen and lymph nodes were calculated and compared between low-dose IL-2 treated and PBS treated mice. (c) Hematoxylin & Eosin staining was performed to show the pathology in lung, liver and kidney on day 8 post LCMV infection. Magnifications were 100X in each image and a randomly selected area was enlarged to 400X in each image. Each dot represents one individual mouse, and results are compiled from three independent experiments with at least four mice per group and bars indicate mean values. Statistical significance was determined by Student t-test. *p<0.05; **p<0.01; NS, not significant different.
(TIF)

S4 Fig Evaluation of tissue immunopathology and tissue infiltration of CD8^+^ T cells in LCMV infected mice with/without low-dose IL-2 treatment. (a) Scoring criteria of the immunopathology and tissue infiltration of CD8^+^ T cells. (b) Immunopathology and tissue infiltration of CD8^+^ T cells in lung, liver, and kidney. Statistical significance was determined by Student t-test. *p<0.05; **p<0.01; NS, not significant different.
(TIF)
