# Supplementary material for: Low-dose IL-2 therapy invigorates CD8+ T cells for viral control in systemic lupus erythematosus
Source: PLoS Pathog. 2021 Oct 7;17(10):e1009858. doi: 10.1371/journal.ppat.1009858 (PMC8525737; doi:10.1371/journal.ppat.1009858)
Supplement: S1 Table — (DOCX) [file ppat.1009858.s001.docx]

**S1 Table. Characteristics of SLE patients after propensity score-matching**

| **Variables** | **Ld-IL-2**  **(n=181)** | **Non-IL-2**  **(n=181)** | ***P* value** |
| --- | --- | --- | --- |
| Gender, Female | 158 (87.3) | 158 (87.3) | 1.000 |
| Age, year | 34 (27, 47) | 33 (27, 48) | 0.890 |
| Duration, year | 7 (3, 12) | 6 (2,11) | 0.251 |
| Comorbidities |  |  |  |
| Nephritis | 53 (29.3) | 44 (24.1) | 0.286 |
| Diabetes mellitus | 8 (4.4) | 8 (4.4) | 1.000 |
| Chronic pulmonary disease | 6 (3.3) | 4 (2.2) | 0.521 |
| Treatments |  |  |  |
| Prednisone | 160 (88.4) | 164 (90.6) | 0.493 |
| Baseline prednisone | 15 (10, 30) | 12.5 (6, 35) | 0.387 |
| < 15 mg/d | 76 (42.0) | 82 (45.3) | 0.525 |
| 15-30 mg/d | 41 (22.7) | 36 (19.9) | 0.521 |
| > 30 mg/d | 43 (23.8) | 43 (23.8) | 1.000 |
| Hydroxychloroquine | 127 (70.2) | 134 (74.0) | 0.412 |
| Cyclophosphamide | 17 (9.4) | 21 (11.6) | 0.493 |
| Mycophenolate mofetil | 73 (40.3) | 65 (35.9) | 0.387 |
| Cyclosporine | 23 (12.7) | 28 (15.5) | 0.450 |
| Tacrolimus | 10 (5.5) | 7 (3.9) | 0.456 |
| Azathioprine | 17 (9.4) | 14 (7.7) | 0.573 |
| SLEDAI-2k | 4 (2, 8) | 4 (2, 6) | 0.295 |
| ≤ 4 points | 97 (53.6) | 97 (53.6) | 1.000 |
| 5-9 points | 48 (26.5) | 53 (29.3) | 0.558 |
| 10-14 points | 30 (16.6) | 30 (16.6) | 1.000 |
| ≥ 15 points | 1 (0.6) | 1 (0.6) | 1.000 |
| Infection | 15 (8.3) | 61 (33.7) | <0.001*** |

Data expressed as median (IQR) and n (%) and compared by Fisher’s exact test or Mann-Whitney *U*-tests. ***P<0.001.

IL-2, interleukin 2; SLEDAI-2k, Systemic lupus erythematosus disease activity index-2000.
