# Supplementary material for: Low-dose IL-2 therapy invigorates CD8+ T cells for viral control in systemic lupus erythematosus
Source: PLoS Pathog. 2021 Oct 7;17(10):e1009858. doi: 10.1371/journal.ppat.1009858 (PMC8525737; doi:10.1371/journal.ppat.1009858)
Supplement: S2 Table — (DOCX) [file ppat.1009858.s002.docx]

**S2 Table. Mouse infection scoring index**

|  | 0 | 1 | 2 | 3  (IMMEDIATE EUTHANASIA) |
| --- | --- | --- | --- | --- |
| Coat Condition | Normal | Rough | Unkempt, very poor condition |  |
| Activity / Body posture | Normal | Isolated from other mice, slightly hunched | Huddled, very hunched, inactive |  |
| Breathing | Normal | Rapid, shallow | Rapid, deep with abdominal effort | Laboured, cyanosis (blue extremities) |
| Dehydration | Nil | Mild | Skin tents when pinched | Skin tents, does not return within 2 seconds, eyes sunken |
| Body condition | Normal | Thin on handling | Obvious loss of fat | Loss of muscle, sunken around spine/abdomen. |
| Weight loss | Nil | Reduced growth | > 15 % of pre-experimental bodyweight | > 20 % of pre-experimental weight |
| HUMANE ENDPOINT: A cumulative score of 4 across all categories or an individual score of 3 in any category. | | | | |
