# Supplementary material for: Low-dose IL-2 therapy invigorates CD8+ T cells for viral control in systemic lupus erythematosus
Source: PLoS Pathog. 2021 Oct 7;17(10):e1009858. doi: 10.1371/journal.ppat.1009858 (PMC8525737; doi:10.1371/journal.ppat.1009858)
Supplement: S3 Table — (DOCX) [file ppat.1009858.s003.docx]

**S3 Table. Key Resources Table**

| **REAGENT or RESOURCE** | **SOURCE** | **IDENTIFIER** |
| --- | --- | --- |
| **Antibodies** | | |
| KLRG-1, Alexa Fluor 488, 53-6.7 | BD | 561619 |
| CD44, Alexa Fluor 488, IM7 | Biolegend | 103016 |
| Foxp3, Alexa Fluor 488, MF-14 | Biolegend | 126406 |
| TNF-a, PE, MP6-XT22 | Biolegend | 506306 |
| CD25, PE-cy7, PC61 | Biolegend | 102016 |
| IFN-g, PE-cy7, XMG1.2 | BD | 557649 |
| Perforin, APC, eBioOMAK-D | eBioscience | 17-9392-80 |
| CD8a, Alexa Fluor 700, 53-6.7 | Biolegend | 100730 |
| CD62L, APC-cy7, MEL-14 | Biolegend | 104428 |
| GranzymB, Pacific Blue, GB11 | Biolegend | 515408 |
| PD-1, Brilliant Violet 421, J43 | BD | 562584 |
| CD107a, Brilliant Violet 421, 1D4B | Biolegend | 121618 |
| CD44, V500, IM7 | BD | 560781 |
| CD4, Brilliant Violet 605, RM4-5 | BD | 100548 |
| CD127, Brilliant Violet 605, A7R34 | Biolegend | 135041 |
| CD3, Brilliant Violet 650, 17A2 | Biolegend | 100229 |
| B220, Brilliant Violet 786, RA3-6B2 | BD | 563894 |
| 7AAD | Thermo Fisher | A1310 |
| CD8a | Servicebio | GB11068 |
| Streptavidin, PE-CF594 | BD | 562318 |
| **Chemicals, Peptides, and Recombinant Proteins** | | |
| 7AAD Staining Solution | BD Biosciences | Cat#559925 |
| Lysing Solution 10X concentrate | BD Biosciences | Cat#349202 |
| RPMI 1640 | Sigma | R0883 |
| FBS | Gibco | Cat#10100147 |
| GP33 peptide, KAVYNFATC | KE Biochem |  |
| NP396 peptide, FQPQNGQFI | KE Biochem |  |
| HRP conjugate | Servicebio | G23303 |
| Foxp3/Transcription Factor Staining Buffer Set | eBioscience | 00-5523-00 |
| Cytofix/Cytoperm | BD | 554714 |
| **Virus Strains** | | |
| Influenza A virus strain A/HKx31 (H3N2) | | |
| lymphocytic choriomeningitis virus (LCMV), Armstrong | | |
| **Critical Commercial Assays** | | |
| Masson’s trichrome staining kit | Servicebio | G1006 |
| Aspartate aminotransferase Activity Assay Kit | Changchun huili |  |
| Alanine aminotransferase Activity Assay Kit | Changchun huili |  |
| Urea nitrogen Detection Kit | Changchun huili |  |
| Creatinine Assay Kit | Changchun huili |  |
| Anti-dsDNA antibody | Orgentec Diagnostika GmbH | 2001934 |
| Complement | Beckman Coulter | 446450 |
